# Fracture risk and survival outcomes of radium-223 therapy for bone metastases in mCRPC and the modulatory role of bone-protective therapy: a systematic review and meta-analysis

**DOI:** 10.3389/fonc.2025.1716931

**Published:** 2026-01-05

**Authors:** Mingzhong Xiao, Fan Liu, Hong Duan

**Affiliations:** 1 School of Clinical Medicine, West China Hospital of Sichuan University, Chengdu, Sichuan, China; 2 School of Clinical Medicine, Ningxia Medical University, Yinchuan, Ningxia, China

**Keywords:** bone metastases, bone-protective agents, castration-resistant prostate cancer, meta-analysis, radium-223

## Abstract

**Background:**

Metastatic castration-resistant prostate cancer (mCRPC) frequently involves the skeleton. While Radium-223 (Ra-223) alleviates symptomatic bone lesions, its effect on overall survival (OS) and fracture risk is debated. Bone-protective agents (BPAs) may play a critical modulatory role. This study systematically examined how Ra-223 influences OS and fracture risk and the effect of concomitant BPA use.

**Methods:**

As per Preferred Reporting Items for Systematic Reviews and Meta-Analyses (PRISMA) guidelines, PubMed, Embase, the Cochrane Library, and Web of Science were retrieved for randomized controlled trials (RCTs) and cohort studies through July 14, 2025. The primary outcomes were OS and fracture incidence in this PROSPERO-registered review (Registration No.: CRD420251102769). Hazard ratios (HRs) with 95% confidence intervals (CIs) were preferentially extracted; relative risks (RRs) were used when necessary. Heterogeneity was assessed using the I² statistic to guide the choice of random-effects versus random-effects models. Leave-one-out sensitivity analyses were conducted, and study quality was appraised using the NIH tool for RCTs and the Newcastle-Ottawa Scale (NOS) for cohort studies.

**Results:**

Nine studies were included, including six RCTs and three cohort studies. The pooled OS analysis (five studies, n=3,671) showed no significant benefit (HR = 0.82, 95% CI: 0.60-1.11; I²=79.8%). Excluding one study on an abiraterone background revealed a survival advantage (HR = 0.71, 95% CI: 0.61-0.81; I²=43.5%). For fracture risk, pooled analysis (five studies, n=3,671) showed no significant increase (HR = 1.32, 95% CI: 0.68-2.58; I²=89.1%). Concomitant BPA use (3 studies, n=279) was associated with a substantial fracture risk reduction (RR = 0.23, 95% CI: 0.11-0.45; I²=0.0%).

**Conclusions:**

Current evidence suggests that Ra-223 administered alongside standard therapy is not associated with statistically significant differences in OS or fracture risk among patients with mCRPC. Concomitant BPA therapy significantly reduces fracture incidence. Therapeutic context, including concurrent therapies and sequencing, may influence survival. Routine evaluation and consideration of BPA use during Ra-223-based regimens, together with strengthened bone health monitoring protocols, are advisable. Given the limited number of eligible studies and substantial heterogeneity, additional high-quality RCTs and individual patient data meta-analyses are needed to clarify these associations.

**Systematic Review Registration:**

https://www.crd.york.ac.uk/prospero/, identifier CRD420251102769.

## 1 Introduction

Prostate cancer (PC), among the most common malignancies in males globally, is the fifth leading cause of cancer-linked death among men (1, 2). Across 185 countries, PC is the predominant malignancy in 112 countries, accounting for over 50% of all cases (3). In 2020, there were 1,414,249 new and 375,000 dead cases arising from PC worldwide (1, 2, 4, 5), with a substantial proportion of metastatic castration-resistant prostate cancer (mCRPC) (6).

The skeleton is the predominant site of metastasis in PC, affecting nearly 10% of individuals with localized disease and up to 80% of those with advanced disease (7, 8). In an autopsy study, 90.1% of men who died from PC with hematogenous spread exhibited osseous metastases (7).

For patients with metastatic PC or postoperative recurrence, androgen deprivation therapy (ADT) remains the standard first-line systemic therapy, with increasing use of combination regimens incorporating abiraterone (9, 10). Individuals newly diagnosed with low-volume metastatic disease may also receive docetaxel (11, 12) or local radiotherapy (13). However, despite these therapies, disease progression commonly leads to mCRPC (14). Bone metastases not only cause severe pain but also impair skeletal integrity and increase the likelihood of symptomatic skeletal events (SSEs) (14).

Radium-223 (Ra-223) is a calcium-mimetic α-emitter that selectively incorporates into newly formed bone matrix within osteoblastic metastases, producing DNA double-strand breaks in adjacent tumor cells, osteoblasts, and osteoclasts (15). In the ALSYMPCA trial, Parker et al. randomized 921 patients with symptomatic mCRPC to Ra-223 or placebo and observed improved overall survival (OS), delayed SSE onset, and better quality of life with Ra-223 (Hazard ratio (HR) =0.70) (16, 17). Nilsson et al. likewise reported delayed time to first bone event with Ra-223 versus placebo (HR = 0.57) (18).

Nevertheless, emerging data suggest a potential increase in fracture incidence associated with Ra-223, particularly in the absence of bone-protective agents (BPAs). In the ERA-223 phase III randomized controlled trial (RCT), Smith et al. noted higher fracture rates in the Ra-223 plus abiraterone group compared with control (29% vs. 11%) without evidence of OS benefit (19). Similarly, the PEACE-3 trial reported a higher fracture incidence in the Ra-223 combination arm (24.3% vs. 13.4%) (20). Conversely, Matsubara et al. observed substantially lower fracture rates when BPAs were used alongside Ra-223 (12.5% vs. 37.5%) (21), and Matsumoto et al. similarly documented reduced fracture incidence with BPA co-administration (4.5% vs. 23.3%) (22).

Therefore, our study endeavors to systematically review and quantitatively evaluate the fracture risk and survival outcomes related to Ra-223 in people having mCRPC and bone metastases, as well as to explore the potential protective role of BPAs. The findings provide critical evidence for oncologists, nuclear medicine specialists, and orthopedic clinicians, while also informing updates to clinical practice guidelines.

## 2 Methodology

This study followed the Preferred Reporting Items for Systematic Reviews and Meta-Analyses (PRISMA) statement (**Supplementary Table S1**) (23). Its protocol was prospectively registered on the PROSPERO platform (Registration No.: CRD420251102769).

### 2.1 Eligibility criteria

Before the initiation of this study, all collaborators collectively established the eligibility criteria using the PICOS framework: Population (P): Adult patients (>18) with histologically confirmed mCRPC and two or more bone metastases via bone scan. Intervention (I): 1) Experimental groups receiving intravenous Ra-223 in addition to standard therapy; 2) patients receiving Ra-223 and BPAs. Comparison (C): 1) Control groups receiving standard therapy without Ra-223, with or without placebo; 2) patients receiving Ra-223 without BPAs. Outcomes (O): OS, fracture risk, and incidence of SSEs. Study design (S): RCTs or cohort studies.

Exclusion criteria were: 1) meta-analyses, reviews, or commentaries; 2) case reports or study protocols; 3) animal studies or *in vitro*/*in vivo* experiments; 4) non-English, duplicate, or retracted articles; 5) studies not meeting PICOS criteria.

### 2.2 Search strategy and sources

PubMed, Embase, Cochrane Library, and Web of Science were retrieved until July 14, 2025. Search terms were determined using controlled vocabulary (MeSH/Emtree) and free-text terms related to mCRPC and Ra-223. Boolean operators (AND/OR) and field restrictions (Title/Abstract/MeSH terms) were applied to refine the search strategy. Taking PubMed as an example, searches using MeSH terms (‘Neoplasm Metastasis[MeSH Terms]’) and free-text terms for tumor metastasis were initially conducted, generating search histories #1 and #2, respectively. Subsequently, analogous searches were performed using MeSH terms (‘Radium[MeSH Terms]’) and free-text terms for radium, yielding search histories #3 and #4. These terms were combined using the Boolean operator strategy: (#1 OR #2) AND (#3 OR #4). The same methodology was applied across all other databases, with full strategies provided in **Supplementary Table S2**.

### 2.3 Study selection

Two independent reviewers (Xiao and Liu) selected the studies. All retrieved references were uploaded to EndNote for duplicate removal. Titles, keywords, and abstracts were checked. Full texts of possibly eligible studies were subsequently reviewed. Dissents were addressed via discussion under the supervision of a third reviewer (Duan).

### 2.4 Data extraction

The following data were independently extracted by Xiao and Liu via a standard form: study characteristics (first author, publication year, country, sample size), demographics (age, sex, Eastern Cooperative Oncology Group (ECOG) performance), and outcome measures (OS, fracture risk, SSEs). Because several studies reported SSEs without separate fracture data, SSEs were treated analytically as fractures, acknowledging fractures as the predominant component of SSEs. For fracture and mortality outcomes, HRs with 95% confidence intervals (CIs) were recorded. In studies reporting only event counts at specific time points, relative risks (RRs) were derived based on the number of events and non-events.

To explore heterogeneity and strengthen methodological rigor, subgroup analyses were conducted among the included RCTs. As an initial refinement, the retrospective study by Stattin et al. (2022) was excluded to reduce selection bias. RCTs were then stratified into three clinically relevant subgroups based on systemic therapy and bone-protection strategies: Group 1: Ra-223 combined with conventional standard therapy; Group 2: Ra-223 plus novel hormonal agents (enzalutamide/abiraterone) with mandatory bone-modifying agents; Group 3: Ra-223 plus novel hormonal agents without required bone-protective measures.

### 2.5 Quality assessment

Xiao and Liu independently rated study quality through standardized tools. For RCTs, the National Institutes of Health (NIH) Quality Assessment Tool for Controlled Intervention Studies was applied, encompassing 14 criteria (https://www.nhlbi.nih.gov/health-topics/study-quality-assessment-tools). Each was scored as “Yes,” “No,” or “Not Reported,” with 1 point awarded only for “Yes.” Studies scoring 0–5 were classified as high risk of bias (Poor), 6–10 as moderate risk (Fair), and 11–14 as low risk (Good). Cohort studies were assessed via the Newcastle-Ottawa Scale (NOS with a maximum of 9 stars)(https://www.ohri.ca/programs/clinical_epidemiology/oxford.asp) and classified according to AHRQ standards: Good (Selection 3–4 stars, Comparability 1–2 stars, Outcome/Exposure 2–3 stars), Fair (Selection 2 stars, Comparability 1–2 stars, Outcome/Exposure 2–3 stars), Poor (any domain below threshold). Dissents were addressed via discussion with a third reviewer (Duan).

Given the inconsistent findings across prior randomized trials, particularly regarding fracture risk and OS, and the heterogeneity observed in preliminary pooled analyses, a formal Grading of Recommendations Assessment, Development and Evaluation (GRADE) evaluation was undertaken to assess the certainty of evidence. Critical outcomes (OS and fracture risk) were evaluated across five domains: risk of bias, inconsistency, indirectness, imprecision, and publication bias. Certainty levels were categorized as high, moderate, low, or very low. Evidence profiles were created using GRADEpro GDT (https://methods.cochrane.org/grading/grade-pro-gdt).

### 2.6 Statistical analysis

Statistical analyses were enabled by Stata 15 (StataCorp, College Station, TX, USA). Heterogeneity was quantified via the I² statistic, with higher values denoting greater heterogeneity. A random-effects model was applied when I² exceeded 50%; otherwise, a fixed-effects model was utilized. Sensitivity analyses were carried out for outcomes with high heterogeneity (I² ≥ 50%) to evaluate the robustness of the results.

## 3 Results

### 3.1 Characteristics of encompassed studies

**Figure 1:**
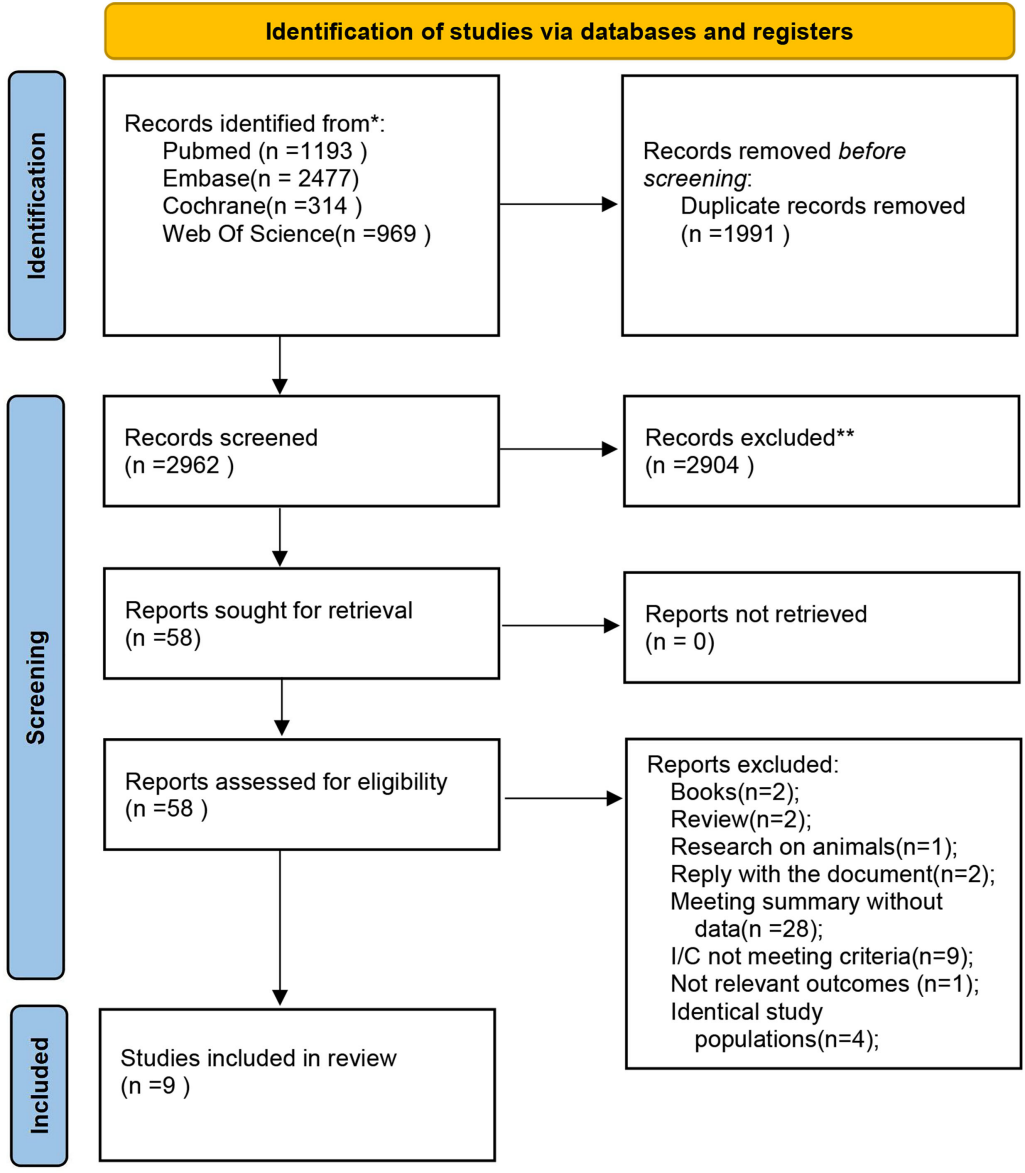
PRISMA flow diagram (PRISMA flow diagram depicting the literature screening process and reasons for inclusion and exclusion.).

4,953 articles were retrieved, of which 1,991 duplicates were removed, leaving 2,962 articles for screening. As per pre-specified eligibility criteria, titles, abstracts, and keywords were initially screened, resulting in the exclusion of 2,904 articles and leaving 58 articles for full-text assessment. Upon obtaining and reviewing the full texts, two were identified as book chapters, two as systematic reviews, one as an animal study, two as correspondence, 28 as conference abstracts lacking data, nine with interventions and comparators not meeting inclusion criteria, one with irrelevant outcomes, and four reporting overlapping populations. Ultimately, nine original studies (16, 18–22, 24–26) were encompassed (**Figure 1**).

### 3.2 Patient demographics

Among nine eligible studies, the mean age was 70-74. Five were multicenter European trials (United Kingdom, Norway, Czech Republic, etc.), two were from Japan, one from Canada, and one from Sweden. Study designs comprised three cohort studies and six RCTs. Regarding tumor metastasis, the most common number of metastatic sites ranged from 6 to 20, with patients in Stattin (2022) primarily exhibiting lymph node metastases. Commonly used medications before enrollment encompassed docetaxel and enzalutamide. Detailed fracture definitions from each original study were also incorporated. In RCTs, particularly ALSYMPCA and earlier phase studies, fractures were typically defined as new symptomatic pathological fractures and analyzed within the broader category of SSEs. After the safety concerns identified in the ERA 223 trial, later studies (e.g., ERA 223, PEACE-3) expanded safety monitoring to include all clinical fractures, with distinctions among pathological, traumatic, and osteoporotic fractures. In contrast, real-world evidence studies generally adopted broader criteria, often using terms such as “pathological fracture” without explicit diagnostic definitions or classifying fractures based on hospitalization requirements. Other baseline characteristics are summarized in **Table 1**.

**Table 1:** Baseline characteristics of encompassed studies.

Study	Country	Study types	Contrast setting	Patients (n)	Prior treatment(n)	Median age (year)	Median PSA ng/ml	Median ALP IU/l	Concomitant BPA n(%)	Histology n(%)	Performance status n(%)	BPI-SF worst pain score, n (%)	Extent of disease n(%)	Primary endpoint	Secondary endpoint	Definition of fracture	Median follow-up (month)	Quality assessment (scores)
Category A Studies																		
Tombal et al(PMID: 40450503) 2025	multinational study	RCT	Enza+Radium-223/ Enza	222 224	denosumab or bisphosphonates(77) docetaxel(67) abiraterone(4) denosumab or bisphosphonates(77) docetaxel(66) abiraterone(7)	70 70	24 21.4	106 124.5	29 (54.7) 17 (56.7)	Gleason score <8 82 (36.9)/73 (32.6) ≥8 138 (62.2)/147 (65.6) Missing 2 (0.9)/4 (1.8)	WHO performance status 0 152 (68.5)/154 (68.8)	Worst pain BPI 0-1 122 (55.0)/121 (54.0) Worst pain BPI 2-3 79 (35.6)/89 (39.7) Worst pain BPI >3 9 (4.1)/10 (4.5) Missing 12 (5.4)/4 (1.8)	N1 stage at randomization 57 (25.7)/52 (23.2) M1b stage at randomization 220 (99.1)/223 (99.6)	rPFS	OS time to subsequent systemic treatment pain progression symptomatic skeletal event	Efficacy Endpoint (SSE): New symptomatic pathologic bone fractures. Safety Endpoint: Fractures occurring during treatment (whether symptomatic or pathologic).	42.3 41.1	NIH-QAT 13
Smith et al(PMID: 30738780) 2019	multinational study	RCT	AAP+Radium-223/ AAP+Placebo	401 405	Docetaxel (9) Ketoconazole (8) Enzalutamide (32) Sipuleucel-T (11) Docetaxel (6) Ketoconazole (4) Enzalutamide (21) Sipuleucel-T (11)	71 71	30 31	129 121	157 (39%) 172 (42%)	Gleason score <8 140 (35)/154 (38) ≥8 246 (61)/233 (58) Missing 15 (4)/3 (1)	ECOG performance status 0 262 (65)/281 (69) 1 137 (34)/121 (30) Missing 3 (1)/3 (1)	0 (asymptomatic) 195 (49)/198 (49) 1–3 (mildly symptomatic) 181 (45)/174 (43) Missing 2 (<1)/3 (1)	Normal or abnormal because of benign bone disease 2 (<1)/0 <6 metastases 134 (33)/141 (35) 6−20 metastases 175 (44)/181 (45) >20 metastases (not superscan) 71 (18)/70 (17) Superscan 19 (5)/13 (3)	SSES	OS time to opiate use for cancer pain time to cytotoxic chemotherapy radiological progression-free survival time to pain safety	Efficacy Endpoint (SSE): New symptomatic pathologic bone fractures. Safety Endpoint: Clinical fractures, including pathological, traumatic, and osteoporotic fractures.	21.2	NIH-QAT 12
Sartor et al(PMID: 24836273) 2014	multinational study	RCT	Best standard of care+Radium-233/ Best standard of care+Placebo	614 307	docetaxel(352) docetaxel(174)	71 71	146 173	211 223	250 (41%) 124 (40%)	NR	ECOG performance status 0 165 (27)/78 (26) 1 371 (61)/187 (61) ≥2 77 (13)/41 (13) WHO ladder for cancer pain 1 257 (42)/137 (45) 2 151 (25)/78 (25) 3 194 (32)/90 (29)	NR	<6 metastases 100 (16)/38 (12) 6−20 metastases 262 (43)/147 (48) >20 metastases (not superscan) 195 (32)/91 (30) Superscan 54 (9)/30 (10)	OS	skeletal-related events(SREs)	New symptomatic pathologic bone fractures. Explicitly states all events must be clinically apparent, not assessed by periodic radiological review.	NR	NIH-QAT 13
Matsubara et al(PMID: 31823152) 2019	Japan	RCT	AAP+Radium-223/ AAP+Placebo	57 57	Enzalutamide (10) Bicalutamide (53) Flutamide (25) History of CAB (51) Enzalutamide (4) Bicalutamide (55) Flutamide (29) History of CAB (54)	73 73	16.06 7.74	288 253	32 37	Gleason score <8 8 (14.0)/11 (19.3) ≥8 49 (86.0)/44 (77.2) Missing 0/2 (3.5)	ECOG performance status 0 40 (70.2)/49 (86.0) 1 17 (29.8)/8 (14.0)	BPI-SF Worst Pain Score, n (%) 0 (asymptomatic) 30 (52.6)/31 (54.4) 1–3 (mildly symptomatic) 27 (47.4)/25 (43.9) Missing 0/1 (1.8)	Normal or abnormal because of benign bone disease 1 (1.8)/0 <6 metastases 19 (33.3)/23 (40.4) 6−20 metastases 24 (42.1)/26 (45.6) >20 metastases (not superscan) 11 (19.3)/6 (10.5) Superscan 2 (3.5)/2 (3.5)	SSE-free survival (SSE-FS)	overall survival (OS)	Efficacy Endpoint (SSE): New symptomatic pathologic bone fracture (requires central review confirmation of bone metastasis at site). Safety Endpoint (AE): Any type of fracture (pathological, traumatic, osteoporotic)	NR	NIH-QAT 11
Stattin et al(PMID: 36180341) 2022	Sweden	Cohort Study	Radium-223/ comparator drugs	681 753	Docetaxel (250) Cabazitaxel (60) Abiraterone (181) Enzalutamide (262) Others (22) Docetaxel (156) Cabazitaxel (22) Abiraterone (111) Enzalutamide (151) Others (13)	74 74	268 191	300 240	230 130	Gleason grade ≤6 80/111 =7 208/255 >7 393/387	ECOG PS, n (%) 0 269/318 1 305/300 2 100/124 3 7/11	NR	Visceral metastasis, n (%) 28(4)/105(14) Lymph node metastasis, n (%) 176(26)/32(43) Other site of metastasis, n (%) 22(3)/43(6) Prior diagnosis of other cancer, n (%) 27(4)/39(5)	Risk of bone fractures	All-cause mortality Prostate cancer–specific mortality	Bone fractures requiring admission to a hospital or treatment in an outpatient setting.	NR	NOS 7
Nilsson et al(PMID: 17544845) 2007	multinational study	RCT	Radium-223/ Placebo	33 31	NR	73 72	167 233	228 279	NR	NR	ECOG performance status 0 9/6 1 18/20 2 6/5	Pain severity index 3.50/4.00	Extent of disease <6 metastases 12/7 6–20 metastases 10/13 >20 metastases 11/11	change in bone-alkaline phosphatase (ALP) concentration time to skeletal-related events(SREs)	toxic effects time to prostate-specifi c-antigen (PSA) progression OS	New pathological bone fractures (vertebral and non-vertebral).	NR	NIH-QAT 12
Parker et al(PMID: 23863050) 2013	multinational study	RCT	Radium-223/ Placebo	614 307	docetaxel (352) docetaxel (174)	71 71	146 173	211 223	250 124	NR	ECOG performance-status score,no (%) 0 257/137 1 151/78 ≥2 194/90	NR	<6 metastases 100/38 6–20 metastases 262/147 >20 metastases 195/91 Superscan 54/30	OS	time to the first symptomatic skeletal event various biochemical end points	New symptomatic pathologic bone fractures (vertebral or nonvertebral).	NR	NIH-QAT 14
Category B Studies																		
Zhang et al(ISSN:0732183X (print)) 2024	Canada	Cohort Study	Radium-223+BPA/ Radium-223	53 39	NR	72 72	NR	NR	53 39	NR	ECOG performance status,no(%) 0-1 57 (62) 2 35 (38)	NR	NR	incidence of pathologic fracture	pain response alkaline phosphatase (ALP) response OS	NA	NR	NOS 8
Matsubara et al(PMID: 31823152) 2019	Japan	RCT	Radium-223+BPA/ Radium-223	32 57	NR	NR	NR	NR	32 0	NR	NR	NR	NR	SSE-FS	OS	Efficacy Endpoint (SSE): New symptomatic pathologic bone fracture (requires central review confirmation of bone metastasis at site). Safety Endpoint (AE): Any type of fracture (pathological, traumatic, osteoporotic)	NR	NIH-QAT 7
Matsumoto et al(PMID: 36305673) 2023	Japan	Cohort Study	Radium-223+BPA/ Radium-223	43 30	ARPI(54) Taxane(16)	73	119	328	NR	Biopsy Gleason score 6 3 7 13 8 18 ≥9 39	ECOG performance 0 51 1 19 2 2 3 1	Pain 31	Extent of disease 0 0 1 56 2 10 3 6 4 1	OS	Maximum decline of ALP, LDH, PSA values The rate of adverse events Time to pathological fracture	Pathological fracture and Non-pathological fracture.	NR	NOS 7

### 3.3 Risk of bias

Among the six RCTs, methodological quality was generally good. Parker (2013) achieved a full score of 14/14, whereas the BPA subgroup of Matsubara (2019) scored 7 points; all other studies scored ≥11. Most trials adequately implemented randomization, allocation concealment, blinding, and outcome assessment, applied intention-to-treat (ITT) analysis, and reported <20% loss to follow-up. Limitations were noted in the description of intervention consistency and reporting of pre-registered outcomes or subgroups (**Supplementary Table S3**). The three cohort studies each scored 8 on the NOS, indicating good quality. Cohorts were representative, non-exposed comparators were appropriately selected, exposures and outcomes were adequately assessed, and follow-up was sufficient. Potential confounding remained in the comparability domain, including incomplete adjustment for prior fracture history, bone metastasis burden, and BPA treatment details (**Supplementary Table S4**).

### 3.4 Presentation of meta-analysis results

#### 3.4.1 OS and fracture

**Figure 2:**
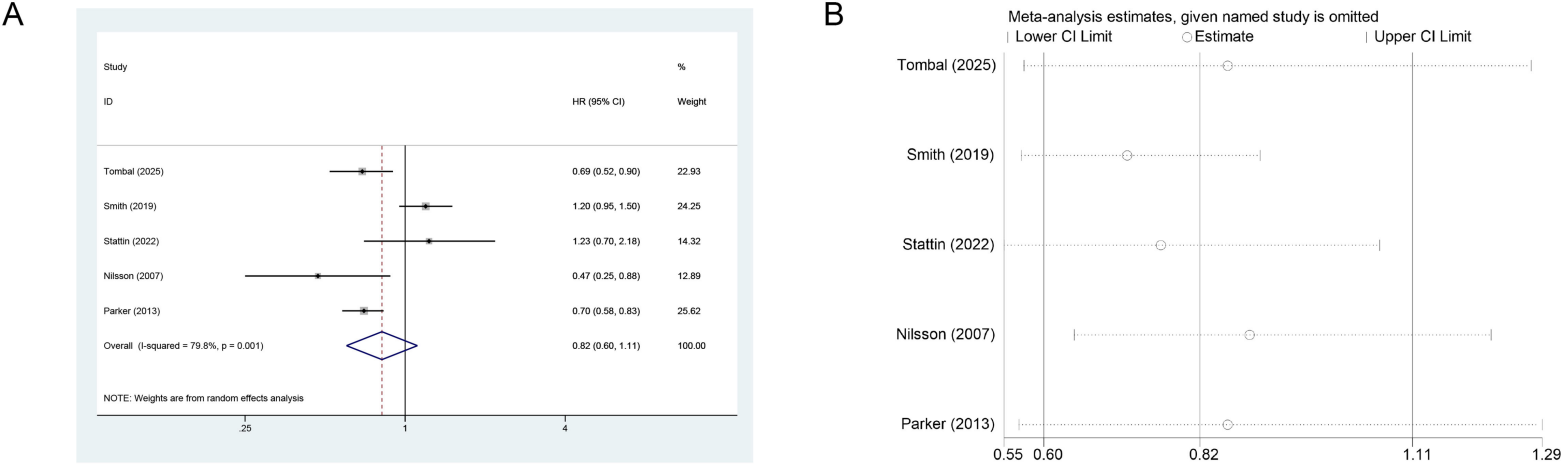
**(A)** Forest plot illustrating the OS impact of Ra-223 in mCRPC patients. The square sizes represent study weights, while the diamond denotes pooled HR. **(B)** LOO sensitivity analysis demonstrating significant reduction in heterogeneity and maintained statistical significance upon exclusion of the Smith study).

Five studies (n=3,671) reported the effect of Ra-223 on OS. Random-effects meta-analysis showed no significant OS benefit (HR = 0.82, 95% CI: 0.60-1.11; I²=79.8%) (**Figure 2A**). Individual studies by Tombal (2025; HR = 0.69, 95% CI: 0.52-0.90), Parker (2013; HR = 0.70, 95% CI: 0.58-0.83), and Nilsson (2007; HR = 0.47, 95% CI: 0.25-0.88) suggested potential survival advantages. Sensitivity analysis using a leave-one-out (LOO) approach confirmed robustness. Excluding Smith (2019) improved the pooled OS estimate (HR = 0.71, 95% CI: 0.61-0.81; I²=43.5%) (**Figure 2B**).

**Figure 3:**
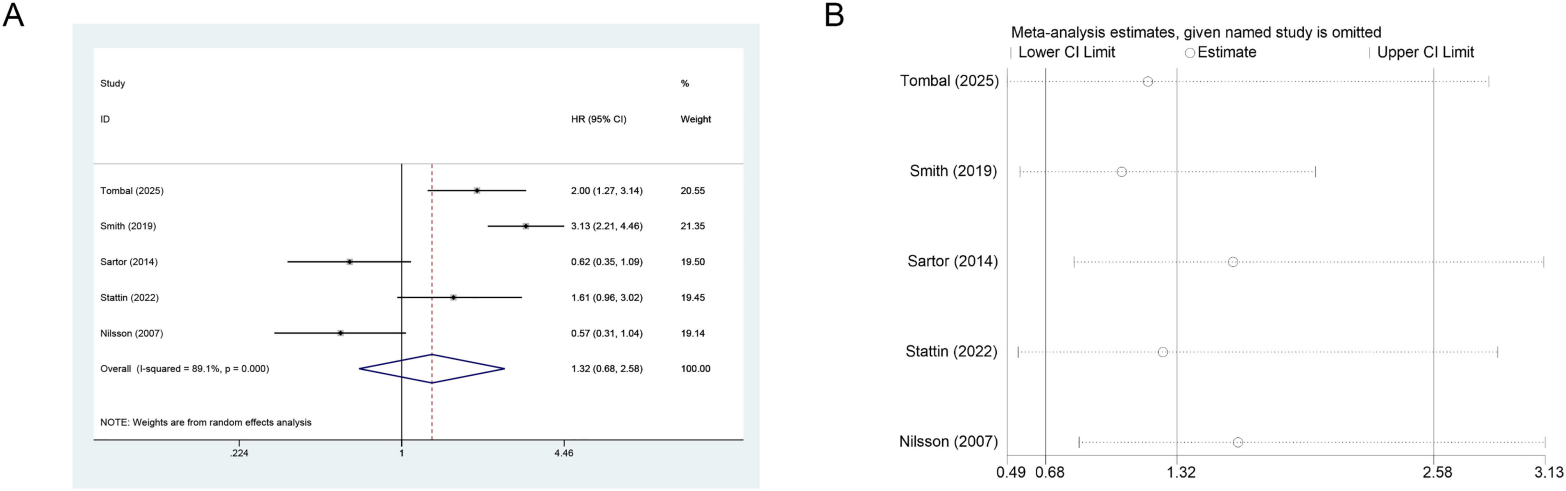
**(A)** Forest plot of ra-223 effects on fracture risk in mCRPC patients. **(B)** Sensitivity analysis of ra-223 effects on fracture risk.

For fracture risk, five studies (n=3,671) showed no statistically significant increase with Ra-223 (HR = 1.32, 95% CI: 0.68-2.58; I²=89.1%) (**Figure 3A**). Tombal (2025; HR = 2.00, 95% CI: 1.27-3.14) and Smith (2019; HR = 3.13, 95% CI: 2.21-4.46) reported elevated risk, whereas other studies found no association. LOO sensitivity analysis confirmed stable results (**Figure 3B**).

#### 3.4.2 BPAs

**Figure 4:**
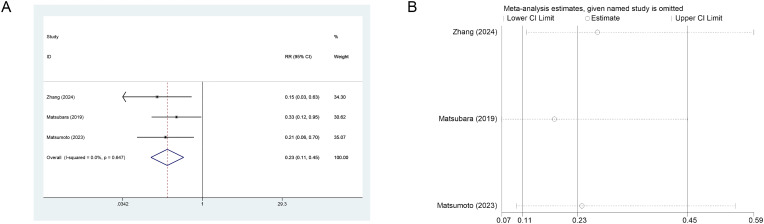
**(A)** Forest plot of BPA effects on fracture risk in mCRPC patients. **(B)** Sensitivity analysis of BPA effects on fracture risk.

Regarding Ra-223 treatment, three studies (n=279) compared the effect of BPA versus no BPA on fracture risk. Results consistently indicated benefit. Fixed-effects meta-analysis demonstrated that BPA significantly reduced fracture risk (RR = 0.23, 95% CI: 0.11-0.45, I²=0.0%) (**Figure 4A**). LOO sensitivity analysis confirmed the robustness of these findings (**Figure 4B**).

**Figure 5:**
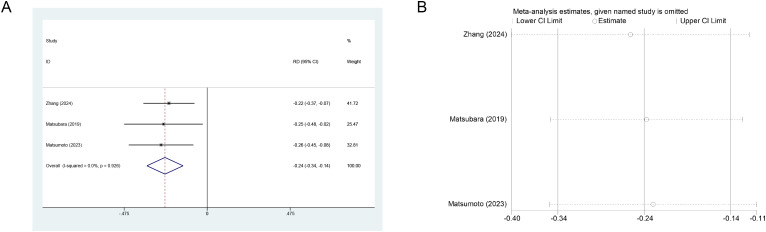
**(A)** Forest plot of the absolute RD for fractures in patients treated with ra−223 with versus without BPAs. **(B)** Leave−one−out sensitivity analysis.

Absolute risk difference (RD) for fractures was evaluated between patients receiving Ra-223 with concomitant BPAs and those without. Pooled analysis demonstrated a significant fracture risk reduction with BPA use (RD=-0.24, 95% CI: -0.34 to -0.14; I²=0.0%) (**Figure 5A**), corresponding to a 24% absolute risk reduction. Results remained robust in leave-one-out sensitivity analysis (**Figure 5B**). The Number Needed to Treat (NNT), calculated as 1/|RD|, was 4.17, indicating that approximately one fracture could be prevented for every four patients treated with Ra-223 plus BPAs.

#### 3.4.3 Subgroup analysis and heterogeneity

For OS, subgroup analysis reduced heterogeneity. Group 1 (Ra−223 with conventional therapy) showed a significant survival benefit (HR = 0.65, 95% CI: 0.47-0.88; I²=29.8%, p=0.233). Divergent outcomes were observed in novel hormonal agent combinations: Group 2 (Ra−223 + novel agents + mandatory BPA) exhibited improved OS (HR = 0.69, 95% CI: 0.52-0.91), whereas Group 3 (Ra−223 + novel agents without mandated BPA) showed no survival advantage (HR = 1.20, 95% CI: 0.95-1.50) (**Supplementary Figure S1**).

Fracture risk demonstrated treatment-dependent variability. Group 3 showed markedly elevated risk (HR = 3.13, 95% CI: 2.21-4.46), Group 2 also exhibited increased risk (HR = 2.00, 95% CI: 1.27-3.14), while Group 1 displayed a protective profile (HR = 0.60, 95% CI: 0.39-0.90) (**Supplementary Figure S2**).

### 3.5 GRADE assessments

Certainty of evidence for OS and fracture risk was rated low (⨁⨁◯◯) due to substantial heterogeneity (I²=79.8% and 89.1%, respectively). Although some studies suggested potential survival benefits or increased fracture risk with Ra-223, inter-study variability persisted, likely reflecting residual confounding. In contrast, short-term (6–16 months) fracture risk analysis showed a significant protective effect of BPAs (RR = 0.23, 95% CI: 0.11-0.45; RD=-0.24, 95% CI: -0.34 to -0.14) with high-certainty evidence (⨁⨁⨁⨁), underscoring their importance during Ra-223 therapy (**Table 2**).

**Table 2:** GRADE evidence profile.

Certainty assessment							No of patients		Effect	Certainty	Importance
No of studies	Study design	Risk of bias	Inconsistency	Indirectness	Imprecision	Other considerations	ADT+radium-223/radium-223+BPA	ADT/radium-223	Relative(95% CI) or absolute (95% CI)		
OS (ADT+radium-223 compared to ADT) (follow-up: range 21.2 months to 42.3 months)											
5	non-randomised studies	not serious	serious^a^	not serious	not serious	all plausible residual confounding would reduce the demonstrated effect	1951 participants	1720 participants	HR 0.82 (0.60 to 1.11)	⨁⨁◯◯ Low^a^	CRITICAL
Fracture (ADT+radium-223 compared to ADT) (follow-up: range 21.2 months to 42.3 months)											
5	non-randomised studies	not serious	serious^b^	not serious	not serious	all plausible residual confounding would reduce the demonstrated effect	1951 participants	1720 participants	HR 1.32 (0.68 to 2.58)	⨁⨁◯◯ Low^b^	CRITICAL
Fracture (radium-223+BPA compared to radium-223) (follow-up: range 6 months to 16 months)											
3	non-randomised studies	not serious	not serious	not serious	not serious	strong association all plausible residual confounding would reduce the demonstrated effect	9/128 (7.0%)	29/151 (19.2%)	RR 0.23 (0.11 to 0.45)	⨁⨁⨁⨁ High	CRITICAL
Fracture (Radium-223+BPA compared to Radium-223) (follow-up: range 6 months to 16 months)											
3	non-randomised studies	not serious	not serious	not serious	not serious	very strong association all plausible residual confounding would reduce the demonstrated effect	9/128 (7.0%)	29/151 (19.2%)	RD -0.24 (-0.34,-0.14)	⨁⨁⨁⨁ High	CRITICAL

CI, confidence interval; HR, hazard ratio; RR, risk ratio; RD, risk difference.

Explanations

^a.^1. The initial pooled analysis showed extremely high heterogeneity: I²=79.8% (>60%) across 5 studies (n=3671); 2. The direction of effects varied: 3 studies (Tombal 2025, Parker 2013, Nilsson 2007) suggested an OS benefit, while 2 studies (Matthew 2019, Stattin 2022) showed no significant benefit.

^b.^1. The initial pooled analysis showed high heterogeneity: I² = 89.1% (>50%) across 5 studies (n=3671); 2. The direction of effects varied: 2 studies (Tombal 2025, Smith 2019) suggested an increased fracture risk, while the remaining 3 studies showed no significant association.

## 4 Discussion

This meta-analysis, based on published data, unraveled the impact of Ra-223 on fracture risk and OS among the mCRPC population. Overall, in view of considerable heterogeneity, Ra-223 combined with standard therapy did not significantly improve OS, nor did it increase fracture risk. After Smith’s study (2019) was excluded, the pooled results demonstrated an OS benefit (Section 3.4.1), suggesting that the treatment context possibly influences effect estimates. Concurrent use of BPAs significantly lowers the fracture risk. Given the small sample size and heterogeneity, these conclusions require further validation.

Regarding Ra-223 and OS, studies by Tombal (2025), Parker (2013), and Nilsson (2007) suggested a survival benefit, whereas Smith (2019) and Stattin (2022) did not observe a significant improvement. These discrepancies likely stem from differences in treatment context and patient composition. In Stattin’s study (2022), Ra-223 was administered across varying lines of therapy, leading to baseline confounding (25). Sequential elimination sensitivity analysis revealed that excluding the study by Smith et al. (ERA-223) rendered the results statistically significant, identifying it as a clear outlier. In this trial, patients received Ra-223 concomitant with abiraterone and prednisone. Prednisone is known to impair bone quality, and concurrent administration with abiraterone may further disrupt the bone microenvironment, potentially negating Ra-223 benefits and increasing adverse event risk. After excluding this study, the remaining evidence aligned with findings from the ALSYMPCA trial, supporting OS benefits. These observations highlight the importance of considering concomitant medications when interpreting Ra-223 efficacy.

Ra-223 is a calcium-mimetic α-emitter that selectively localizes in newly formed bone matrix of osteoblastic lesions and induces DNA double-strand breaks in adjacent cells (15). Compared with β-emitters, Ra-223 exhibits shorter tissue penetration (<100 μm) and lower myelotoxicity (27–29), theoretically reducing skeletal adverse event burden. Regarding fractures, Tombal (PEACE-3, enzalutamide background) and Smith (ERA-223 experiment, abiraterone-based treatment) reported increased risk (20), whereas most other studies did not. This may relate to androgen receptor pathway inhibition and androgen deficiency, which reduce bone mass and disrupt microarchitecture (30–33), compounded by variations in BPA use. Heterogeneous fracture definitions across trials further complicate pooled analyses and may contribute to outcome variability. Overall, these meta-analytic findings are consistent with Ra-223’s short-range α-emission and low myelotoxic profile, showing no significant increase in fracture risk.

Bisphosphonates and denosumab are the most commonly used BPAs. Bisphosphonates selectively adsorb to bone surfaces and, once taken up by osteoclasts, inhibit their activity, restoring bone remodeling balance and reducing microarchitectural degradation (34–36). Denosumab inhibits osteoclast formation and survival by blocking the RANKL-RANK signaling pathway, thereby decreasing bone resorption and increasing bone density (37, 38). Within the evidence framework encompassed in this study, concurrent use of BPAs with Ra-223 was related to a marked decrease in fracture risk, supporting the biological rationale for a parallel “antitumor therapy plus bone health management” strategy. Nevertheless, as BPA data are primarily from observational studies where patient selection may correlate with fracture risk, these findings demonstrate associations rather than causality. Potential selection bias was carefully considered, and no premature conclusions regarding BPA’s protective effect were drawn.

Subgroup analyses highlight BPA’s critical role in Ra-223 therapy and identify sources of heterogeneity. After excluding retrospective studies (Stattin et al.), treatment protocol divergence became evident. Ra-223 combined with endocrine therapy consistently elevated fracture risk across groups, but survival outcomes varied. In Group 3 (Smith et al.), absence of mandated BPAs led to high fracture risk (HR = 3.13), likely compromising functional status and negating antitumor efficacy, yielding no survival benefit. In contrast, Group 2 (Tombal et al.) implemented compulsory BPA protocols and maintained significant survival improvement (HR = 0.69) despite increased fracture risk, suggesting that effective bone-targeted therapy allows Ra-223’s antitumor synergy with endocrine therapy to translate into meaningful survival gains.

For patients with mCRPC and bone metastases who are planned for or receiving Ra-223, routine consideration of concurrent bone-protective therapy is recommended, provided no contraindications exist and resources allow. This is particularly important for high fracture-risk populations, including those with prior fragility fractures, low bone mass/osteoporosis, long-term or combination hormone therapy, or elevated fall risk. Baseline and follow-up bone health assessments (e.g., bone mineral density [BMD], calcium/vitamin D supplementation, fall risk management, exercise/rehabilitation guidance) should be reinforced, with interdisciplinary collaboration among oncology, nuclear medicine, orthopedics, and rehabilitation, and dynamic adjustment according to concurrent endocrine regimens and individual patient risk.

Our limitations involve the small number of studies, uneven sample sizes, and substantial heterogeneity in treatment lines, concomitant medications, doses, and follow-up durations, limiting external generalizability. Observational studies may be subject to residual confounding and immortal time bias. Variability in fracture assessment and reporting of BPA exposure (type, dose, adherence) restricted detailed subgroup and dose-response analyses. As fewer than 10 studies were encompassed for each outcome, formal evaluation of publication bias (e.g., Egger/Begg tests) was not performed.

Due to the absence of individual patient data, it was impossible to account for the competing risk of death in our fracture analysis, which may affect the interpretation of results. In the high-mortality context of mCRPC, death is a significant competing event that can preclude fracture occurrence by shortening survival. Without appropriate adjustment, reliance on crude fracture rates may introduce confounding by survival time, potentially obscuring the true biological fracture risk. To address this, future research should advance systematically on several fronts. First, clinical trials should incorporate competing risk analysis to more accurately capture fracture outcomes and quantify the burden of skeletal events. Second, high-quality, prospective RCTs are needed to clarify Ra-223’s effects on OS, fractures, and symptomatic skeletal events across different therapy lines and combination regimens. Concurrently, meta-analyses of individual patient data (IPD) should adjust for key confounders, such as prior fractures, bone mineral density, history of falls, calcium/vitamin D supplementation, and type/adherence of BPAs, and develop risk stratification models. Building on this foundation, further studies should compare the efficacy, cost-effectiveness, and safety profiles of bisphosphonates versus denosumab in reducing fractures during Ra-223 therapy. Finally, long-term, real-world follow-up on quality of life, functional outcomes, and delayed adverse events is essential to establish a robust, high-quality evidence base.

## 5 Conclusion

Current evidence indicates that Ra-223 combined with standard therapy does not confer a statistically significant impact on OS or fracture risk in patients with mCRPC. However, concomitant BPA use significantly reduces fracture incidence. Clinical practice should adopt an integrated management strategy emphasizing both antitumor therapy and bone protection, with individualized decision-making and follow-up based on fracture risk and concurrent treatments.

## Data Availability

The original contributions presented in the study are included in the article/**Supplementary Material**. Further inquiries can be directed to the corresponding author.
